# The Thames and the Sewage

**Published:** 1896-11-21

**Authors:** 


### THE THAMES AND THE SEWAGE.

### A TRIP ON THE "BEATRICE."

*(Continued from page 117.)*

At the time of our visit the outflow from the filter was a clear running stream, with a little green conferva growing at the sides just as may be seen in many a country brook.

Now, what these experiments have proved is that, operating on the large scale, a filter can be maintained in action without any deterioration if only it is so worked that it shall be supplied with air in proportion as it is supplied with sewage. This is the explanation of the failure of many sewage farms and of many attempts at sewage filtration, and at the same it is the secret of the success of the "experimental acre" at Barking. There seems to be no doubt that what happens is that, as the result of the alternate supply of air and sewage, the whole mass of the filter becomes charged with nitrifying microbes (which flourish best in the presence of oxygen and organic nitrogen), to the practical exclusion of the organisms of putrefaction. The result is that when the sewage is turned on it finds every pore of the filter charged with air and every particle of it infected with nitrifying microbes. A vast process of fermentation is at once set up. This is just the converse of what happens in brewing. The brewer adds yeast to the wort, while at Barking they turn the wort, which in this case is the sewage effluent, into a vast fermenting tun loosely filled with porous coke, which is leavened throughout with the necessary microbe. In each case the microbe undergoes enormous growth in the presence of its proper food, and effects enormous changes in the fermentable material around it: in the one case producing alcohol and carbonic acid from its action on the sugar; in the other, combining the nitrogenous materials in the sewage with the oxygen in the filter, and producing nitric acid and ultimately nitrates.

Sewage is not an altogether sweet material to begin with, although not so bad when fresh as many people think; but as it lodges in the settling tanks it tends to become foul, and the sludge which deposits during that lodgment is very foul indeed.

No argument, then, is needed to show the great advantage which would accrue if it were possible to do away with all the present system of liming and precipitating, with the settling-tanks, the sludge, and the sludge boats, and to concentrate the whole business of sewage disposal on a process of filtration so arranged as not merely to filter out the foul ingredients, but to turn them into innocuous sand and salt. Nor is such a consummation entirely visionary. If it be possible by such means, as from the report of Mr. Dibdin to the County Council it clearly is, to clarify sewage and to remove about 80 per cent. of its impurities, converting them into harmless salts, the solution of the sewage problem lies in a nutshell.

Whatever may be the virtues of sewage farms wher from the conformation of the land and the character of the soil, the conditions are favourable, there are circumstances in which the difficulties in the way of any such schemes appear almost insuperable. It must then be a matter of great satisfaction to be assured that the investigations carried on by the officials of the County Council have been so far rewarded that the problem has been practically solved. This is not mere theory, but is the outcome of the practical and successful working of the "experimental acre" during a long period, including the severe winter weather at the beginning of 1895, when, although the surface of the filter froze in parts, the sewage still found its way in without any difficulty.

The great aim, of course, is so to use the filter that it shall ferment the foulness out by a biological process—not merely strain it out to rot and cause a nuisance. Some investigations were on foot, we found, with the object of discovering the particular microbe by which the work was done, and no doubt when it becomes possible to inoculate the filters with a pure culture of this organism much time will be saved in bringing them into a state of full activity, but this is not a matter on which the success of the filters in any way depends, for it is clear that so long as the filtering medium is well aerated and allowed occasional days of rest it is quite capable of charging itself with the ferment necessary to destroy the sewage.

After traversing a couple of malodourous mud boats —fine sea boats no doubt, but unpleasant as to cargo —we found the "Beatrice" waiting for us, and were not sorry to partake of the contents of a vast brown teapot which forms part of her equipment. Meanwhile, the same system of analysing samples of the river water was again carried on until we reached the Hungerford Pier, where we said good-bye to our courteous hosts, having much enjoyed our trip on the "Beatrice," a trip which to them, however, had been a day of work, for indeed they had been, as Mr. Dibdin said at first they would be, "pretty busy" all the way.

